# Management of suspected acute heart failure dyspnea in the emergency department: results from the French prospective multicenter DeFSSICA survey

**DOI:** 10.1186/s13049-016-0300-x

**Published:** 2016-09-17

**Authors:** Tahar Chouihed, Stéphane Manzo-Silberman, Nicolas Peschanski, Sandrine Charpentier, Meyer Elbaz, Dominique Savary, Eric Bonnefoy-Cudraz, Said Laribi, Patrick Henry, Nicolas Girerd, Faiez Zannad, Carlos El Khoury

**Affiliations:** 1 Emergency Department, University Hospital of Nancy, Nancy, France; 2 INSERM, Centre d’Investigations Cliniques 9501, Université de Lorraine, CHU de Nancy, Institut Lorrain du Cœur et des Vaisseaux, Nancy, France; 3 INI-CRCT (Cardiovascular and Renal Clinical Trialists) F-CRIN network, Nancy, France; 4 INSERM UMR-S 1116, Université Lorraine Nancy I, Nancy, France; 5 Department of Cardiology, Lariboisière Hospital, Paris, France; 6 INSERM UMR-S 942, Université Paris-Diderot, Sorbonne Paris Cité, Paris, France; 7 Emergency Department, University Hospital of Rouen, Rouen, France; 8 University of Rouen-Normandy, INSERM UMR-U1096, Rouen, France; 9 Emergency Department, Rangueil University Hospital, Toulouse, France; 10 INSERM, U1027, Toulouse, France; 11 Université Toulouse III – Paul Sabatier, Toulouse, France; 12 Department of Cardiology, Rangueil Hospital, Toulouse, France; 13 Emergency Department and Intensive Care Unit, Annecy-Genevois, Metz-Tessy, France; 14 Department of Cardiology, Hôpital Cardiologique de Lyon, Lyon, France; 15 Emergency Medicine Department, University Hospital of Tours, Paris, France; 16 Emergency Department and RESCUe Network, Lucien Hussel Hospital, Vienne, France; 17 Univ. Lyon, Claude Bernard Lyon 1 University, HESPER EA 7425, Lyon, France

## Abstract

**Background:**

An appropriate diagnostic process is crucial for managing patients with acute heart failure (AHF) in emergency department (ED). Our study aims to describe the characteristics and therapeutic management of patients admitted to the ED for dyspnea suspected to have AHF, their in-hospital pathway of care and their in-hospital outcome.

**Methods:**

Consecutive patients admitted in 26 French ED for dyspnea suspected to be the consequence of AHF, prior to in hospital diagnostic test, were prospectively included at the time of their admission in the DeFSSICA Survey. Clinical characteristics at admission were recorded by the ED physicians. At discharge from ED, patients were categorized as AHF or non-AHF based on the final diagnosis reported in the discharge summary. The completeness of the data was controlled by the local investigator.

**Results:**

From 16/6/2014 to 7/7/2014, 699 patients were included, of whom 537 (77 %) had a final diagnosis of AHF at discharge. Patients with AHF were older (median 83 vs 79 years, *p* = 0.0007), more likely to have hypertension (71 % vs 57 %, *p* = 0.002), chronic HF (54 % vs 37 %, *p* = 0.0004), atrial fibrillation (45 % vs 34 %, *p* = 0.02) and history of hospitalization for AHF in the previous year (40 % vs 18 %, *p* < 0.0001) when compared to patients without AHF. Furosemide and oxygen were used in approximately 2/3 of the patients in the ED (respectively 75 and 68 %) whereas nitrates were in 19 % of the patients. Diagnostic methods used to confirm AHF included biochemistry (100 %), pro-B-type natriuretic peptide (90 %), electrocardiography (98 %), chest X-ray (94 %), and echography (15 %) which only 18 % of lung ultrasound.

After the ED visit, 13 % of AHF patients were transferred to the intensive care unit, 28 % in cardiology units and 12 % in geriatric units. In-hospital mortality was lower in AHF vs non-AHF patients (5.6 % vs 14 %, *p* = 0.003).

**Discussion:**

DeFSSICA, a large French observational survey of acute HF, provides information on HF presentation and the French pathway of care. Patients in DeFSSICA were elderly, with a median age of 83 years. Compared with the French OFICA study, patients in DeFSSICA were more likely to have hypertension (71 % vs 62 %) and atrial fibrillation (45 % vs 38 %). As atrial fibrillation and a rapid heart rate have been closely linked to mortality, detection of atrial fibrillation should be considered systematically.The limited use of nitrates in DeFSSICA may be related to the median SBP of 140 (121–160) mmHg. However, our use of nitrates was similar to those in the EAHFE (20.7 %) and OPTIMIZE-HF (14.3 %) registries. In line with guidelines, the proportions of patients who underwent ECG, biological analysis, or chest X-ray were all >90 % in DeFSSICA. Similarly, BNP or pro-BNP was measured in 93 % of patients, compared with 82 % of patients in the OFICA study. Although BNP may be helpful when the diagnosis of HF is in doubt, ultrasound remains the gold standard. The use of ultrasound in the ED has been reported to accelerate the diagnosis of HF and the initiation of treatment, and shorten the length of stay. In-hospital mortality of HF patients in DeFSSICA was 6.4 %, slightly lower than in the OFICA study (8.2 %). Improved interdisciplinary cooperation has been highlighted as a key factor for the improvement of HF patient care.

**Conclusions:**

DeFSSICA shows that patients admitted for dyspnea suspected to be the consequence of AHF are mostly elderly. The diagnosis of AHF is difficult to ascertain based on clinical presentation in patients with dyspnea. Novel diagnostic techniques such as thoracic ultrasound are warranted to provide the right treatment to the right patients in the ED as early as possible.

**Electronic supplementary material:**

The online version of this article (doi:10.1186/s13049-016-0300-x) contains supplementary material, which is available to authorized users.

## Background

Heart failure (HF) has been estimated to affect approximately 2 % of adults in developed countries [1], and 9 % of those aged 80–89 years [2]. HF is the cause of over 150,000 hospitalizations in France each year, and the costs of treating patients with HF have been estimated to consume around 1 % of the total healthcare costs [2]. Patients with HF often present in acute or subacute decompensation in acute HF, but various other conditions can also cause dyspnea, raising the problem of differential diagnosis.

### Importance

An appropriate diagnostic process is crucial for starting the patient on the right care pathway and to avoid loss of time in care. However, no trial or survey has described the current management of acute HF syndromes in the French emergency medical system.

### Goals of this investigation

The main objective was to assess the diagnostic and therapeutic management of emergency patients with suspected heart failure dyspnea. Secondary objectives were to define the pathway of care according to the Emergency Department (ED) diagnosis and evaluate mortality.

## Methods

### Study design and setting

DeFSSICA (Description de la Filière de Soins dans les Syndromes d’Insuffisance Cardiaque Aigue) is a French prospective survey that recruited consecutive patients presenting with suspected heart failure dyspnea in 26 emergency departments (EDs) in academic hospitals and community and regional hospitals. The study was promoted by the French Society of Cardiology (Société Française de Cardiologie [SFC]), the French Society of Emergency Medicine (Société Française de Médecine d'Urgence [SFMU]) and RESCUe (an emergency cardiovascular network).

DeFSSICA received approval from the National Commission for Liberties and Data Protection (Commission Nationale de l’Informatique et des Libertés [CNIL]) (number DR-2014-543) and the Advisory Committee on the Treatment of Information in the field of Health Research (Comité Consultatif sur le Traitement de l’Information en matière de Recherche dans le domaine de la Santé [CCTIRS]) (number 14-291). All patients received written information about the survey objectives.

### Selection of participants

Consecutive patients aged above 18 years admitted to the ED with dyspnea compatible with acute HF were included in the survey by the emergency physician on charge and prior to chest X-ray and laboratory test. Dyspnea compatible with HF was defined as dyspnea associated with peripheral edema and/or pulmonary crackles and/or excessive weight gain and/or use of furosemide.

### Methods and measurements

Data concerning baseline characteristics, medical history, social factors, in-hospital diagnostic tests and treatment, final diagnosis, destination after ED discharge, and in-hospital mortality and length of stay were recorded by emergency physicians in a case report form (CRF). The CRF was structured according to the progress of care. Cardiac sonographic evaluations were judged at the sole discretion of emergency physician. Abnormal chest ray was defined by the presence of cardiomegaly and/or alveolar edema and/or interstitial opacity and/or pleural effusion. The choice of treatment was at the emergency physician’s discretion, according to their usual practice. Final diagnosis of acute HF was retained by emergency physician as a combination of a clinical history, an abnormal chest x-ray, an elevated BNP/proBNP and ultrasound signs. Data were entered into a secured database located at the RESCUe Coordination Center.

Local investigators monitored the data to check for any errors or inconsistencies. They were also in charge of trying to recover missing data. At discharge from ED, patients were categorized as AHF or non-AHF based on the final diagnosis reported in the discharge summary. A hotline staffed by a clinical research assistant was dedicated to the survey (during the daytime).

### Outcomes

This study examined the pathway of care (from transportation to the ED to discharge destination); the use of various diagnostic methods (biological and imaging) and treatments; clinical signs and symptoms; causes of HF; and mortality.

### Analysis

All patients with suspected heart failure dyspnea were included. Comparisons between those with and without a final diagnosis of HF were undertaken. Data are medians and interquartile ranges (IQRs) for continuous variables, and numbers and percentages for qualitative variables. Comparative analyses were performed using the *χ*^2^ or Fisher’s test for binary variables and the Wilcoxon test for analysis of variance for continuous variables. Differences were considered to be statistically significant when the *P* value was <0.05. Analysis were performed using the R statistical package.

## Results

### Characteristics of study subjects

Between June 16 and July 7, 2014, DeFSSICA recorded 699 cases of suspected cardiac dyspnea, of whom 537 (77 %) were ultimately identified as having HF. During the same period, 64,281 emergency visits were recorded, thus HF accounted for 0.8 % of ED visits.

Thirteen (50 %) investigators centers were academic hospitals, 11 (42 %) were community hospitals and 2 (8 %) of them were regional hospitals. The academic hospitals included 349 (50 %) patients, community hospitals included 243 (35 %) patients and regional hospitals included 107 (15 %).

Baseline characteristics of patients with and without an ED diagnosis of HF are shown in Table 1. HF patients were older than those without HF, and just under half of each group were male. HF patients were significantly more likely to have hypertension, chronic heart failure, and atrial fibrillation than patients without HF, but the prevalence of other comorbidities did not differ between the two groups. HF patients were more likely to be taking furosemide, angiotensin-converting enzyme inhibitors (ACEIs) or angiotensin II receptor blockers (ARBs), β-blockers, anticoagulants, and insulin than those without HF. Compared to those without HF, those with HF were more likely to have been hospitalized for HF at least once during the past year; they were also more likely to be under the care of a cardiologist.

**Table 1:** Baseline characteristics of patients with suspected cardiac dyspnea^a^

	HF (*n* = 537)	Not HF (*n* = 158)	*P*
Age, y	83 (77–88)	79 (67–88)	.00069
Men	235 (44)	76 (48)	.38
Comorbidities			
Hypertension	378 (71)	90 (57)	.0015
Chronic heart failure	288 (54)	59 (37)	.00035
Atrial fibrillation	239 (45)	54 (34)	.023
Coronary heart disease	161 (30)	37 (23)	.12
Diabetes mellitus	152 (28)	37 (23)	.18
Chronic renal failure	120 (28)	30 (19)	.41
Chronic respiratory failure	92 (17)	32 (20)	.43
Known valvular disease	99 (19)	22 (16)	.22
Prior medications			
Furosemide	315 (59)	67 (43)	.00059
ACEI/ARB	242 (45)	52 (36)	.011
β-blocker	231 (43)	44 (28)	.0010
Anticoagulant	236 (44)	48 (31)	.0038
Aspirin	163 (31)	40 (26)	.28
Other antiplatelet	61 (11)	16 (10)	.80
Oral antidiabetic	71 (13)	19 (12)	.83
Insulin	71 (13)	10 (6.3)	.028
Amiodarone	58 (11)	19 (12)	.74
Aldosterone antagonist	39 (7.3)	11 (6.9)	.94
Digoxin	41 (7.6)	8 (5.1)	.36
Thiazidine	34 (6.3)	12 (7.6)	.68
None	29 (5.4)	19 (12)	.0060
Unknown	13 (2.4)	7 (4.4)	.28
Pacemaker			
Single	18 (3.3)	4 (2.5)	.81
Dual	39 (7.3)	9 (5.7)	.63
Triple	7 (1.3)	1 (0.6)	.80
Defibrillator	17 (3.2)	1 (0.6)	.14
Prior hospitalization for HF during past year			
0	301 (60)	123 (82)	<.0001
1	136 (27)	17 (11)	.00011
≥ 2	67 (13)	10 (6.6)	.039
Followed by a cardiologist	367 (72)	92 (60)	.0090
Residence			.54
At home	445 (83)	124 (79)	
Retirement institution	82 (15)	30 (19)	
Other institution	8 (1.5)	3 (1.9)	
Self-sufficient	273 (52)	98 (64)	.012
Home assistance			
Housekeeper	157 (30)	27 (18)	.0036
Family support	138 (26)	41 (27)	.99
Nurse	129 (24)	21 (14)	.0060
Known cognitive impairment	85 (16)	24 (15)	.88
Bedridden	48 (8.99)	18 (12)	.45

*ACEI* angiotensin-converting enzyme inhibitor, *ARB* angiotensin II receptor blocker, *HF* heart failure, *IQR* interquartile range

^a^Data are presented as n (%) or median (IQR). All percentages are out of patients with non-missing data

Most patients, with or without HF, were living at home (Table 1). However, patients with HF were less likely to be self-sufficient, and more likely to be in receipt of home assistance.

### Hospitalization and clinical status

Among all DeFSSICA patients, 63 % made their own way to the ED (Table 2). For the remaining patients, medical dispatch centers (Centres 15 or services d’aide médicale urgente [SAMUs]) mobilized appropriate prehospital assistance. There was no difference in the distribution of transport modes between patients with and without HF.

**Table 2:** Hospitalization route and clinical status of patients with suspected cardiac dyspnea^a^

	HF (*n* = 537)	Not HF (*n* = 158)	*P*
Means of transport			
Personal	342 (64)	99 (63)	.81
Ambulance	91 (17)	30 (19)	.35
Firemen	55 (10)	17 (11)	.85
MICU	43 (8)	11 (7)	.67
Inter-hospital transfer	6 (1)	1 (0.6)	.59
Clinical signs			.62
Warm extremities	414 (87)	124 (85)	
Cold extremities	62 (13)	22 (15)	
Signs of right heart failure	228 (43)	31 (20)	<.0001
Inspiratory retraction	154 (29)	36 (23)	.18
Inability to speak	45 (8.4)	11 (6.9)	.67
First recorded vital signs			
Heart rate, beats/min	85 (71–102)	81 (70–102)	.41
SBP, mmHg	140 (121–160)	138 (118–155)	.16
DBP, mmHg	75 (65–90)	74 (65–90)	.43
SBP <100 mmHg	38 (7.0)	12 (7.6)	.96
Respiratory rate, breaths/min	25 (20–30)	22 (19–30)	.25
Pulse oximetry, %	94 (90–96)	95 (91–98)	.032
GCS	15 (15–15)	15 (15–15)	.89
Temperature, °C	37 (36–37)	37 (36–38)	.052
Killip status			<.0001
1	133 (25)	90 (59)	
2	283 (55)	45 (29)	
3	92 (18)	14 (9)	
Signs of shock	17 (3.2)	4 (2.53)	

*DBP* diastolic blood pressure, *GCS* Glasgow Coma Scale, *HF* heart failure, *IQR* interquartile range, *MICU* mobile intensive care unit, *SBP* systolic blood pressure

^a^Data are presented as n (%) or median (IQR)

Clinical signs were mainly similar between patients with and without HF, but HF patients were more likely to present with signs of right heart failure (Table 2). Vital signs were also mainly similar between patients with and without HF, although pulse oximetry was lower among those with HF. Only 3.2 % of patients with or without HF presented signs of cardiogenic shock, while 54 and 18 % of HF patients were Killip 2 or 3, respectively, versus 29 and 9.4 %, respectively of patients without HF (*P* < .0001).

### Early management and diagnosis

At admission, all patients (100 %) underwent biological analysis. Patients with HF had lower creatinine clearance and higher B-type natriuretic peptide (BNP) and pro-BNP (Table 3). Troponin was positive in 66 % of HF patients versus 46 % in non-HF patients (*P* = .0001).

**Table 3:** Biological and diagnostic tests^a^

	HF (*n* = 537)	Not HF (*n* = 158)	*P*
Biological analysis			
Performed	537 (100)	158 (100)	1
Sodium, mmol/L	138 (135–141)	138 (136–140)	.69
Potassium, mmol/L	4.3 (4.0–4.7)	4.2 (3.9–4.7)	.092
Creatinine clearance, mL/min	50 (35–69)	65 (42–92)	<.0001
Creatinine clearance <30 mL/min	89 (17)	19 (14)	.39
Hemoglobin, g/dL	12.4 (11.0–13.5)	13.2 (11.5–14.3)	.15
Hemoglobin <10 g/dL	56 (11)	14 (8.9)	.70
Troponin positive	282 (66)	56 (46)	.00010
BNP,^b^ ng/L	973 (502–2414)	338 (129–654)	<.0001
Pro-BNP,^c^ ng/L	4025 (1729–8863)	1281 (283–4818)	<.0001
ECG			
Performed	530 (99)	151 (96)	.018
Sinusal	231 (44)	84 (56)	.012
Atrial fibrillation	226 (43)	41 (27)	.00073
Driven	48 (8.9)	15 (9.5)	.87
AVB	23 (4.3)	12 (7.6)	.12
LBBB	91 (17)	12 (7.6)	.0076
RBBB	62 (12)	20 (13)	.71
Repolarization disorder	107 (20)	32 (21)	.88
Chest X-ray			
Performed	511 (95)	129 (88)	.0023
Abnormal	481 (95)	98 (71)	<.0001
Cardiomegaly	252 (52)	51 (52)	.96
Interstitial opacities	300 (62)	46 (47)	.0047
Alveolar opacities	117 (24)	36 (37)	.019
Echography			
Performed	85 (16)	18 (11)	.20
By cardiologist	48 (56)	13 (65)	.33
Satisfactory^d^	24 (60)	10 (91)	
Intermediate^d^	14 (35)	1 (9.1)	
Weak^d^	2 (5.0)	0 (0)	
By emergency physician	37 (44)	5 (35)	.33
Satisfactory^d^	6 (17)	2 (50)	
Intermediate^d^	20 (57)	2 (50)	
Weak^d^	9 (26)	0 (0)	
LVEF^e^			.93
> 50 %	32 (41)	6 (43)	
35–50 %	27 (34)	4 (29)	
< 35 %	21 (26)	4 (29)	
Dilated RV, n (%)	19 (32)	4 (36)	.94
VC diameter, mm	20 (15–22)	15 (10–20)	.38

*AVB* atrioventricular block, *BNP* brain natriuretic peptide, *ECG* electrocardiogram, *HF* heart failure, *IQR* interquartile range, *IVC* inferior vena cava, *LBBB* left bundle branch block, *LVEF* left ventricular ejection fraction, *RBBB* right bundle branch block, *RV* right ventricle, *US* ultrasound

^a^Data are presented as n (%) or median (IQR) and are among patients who underwent the relevant test and had non-missing data

^b^BNP was measured in 170 HF and 49 not HF patients

^c^Pro-BNP was measured in 329 HF and 74 not HF patients

^d^Self evaluation

^e^LVEF was measured in 80 HF and 14 not HF patients

Most patients (98 %) underwent an ECG. HF patients were more likely to have atrial fibrillation and left bundle branch block (LBBB) (Table 4). The majority of patients underwent chest X-ray (94 %), which was more often abnormal in HF patients (95 % vs 71 %; *P* < .0001).

**Table 4:** Emergency treatment of patients with suspected cardiac dyspnea^a^

	HF (*n* = 537)	Not HF (*n* = 158)	*P*
Furosemide	395 (75)	45 (29)	<.0001
Oxygen	360 (68)	68 (43)	<.0001
Nitrates	101 (19)	11 (6.9)	.00043
Anticoagulant	38 (7.0)	18 (11)	.13
NIV/Boussignac CPAP	54 (10.0)	6 (3.8)	.20
Antiarrythmics	25 (4.7)	8 (5.0)	.96
Inotropic agents	3 (0.6)	1 (0.3)	.61
Tracheal intubation	1 (0.2)	1 (0.3)	.95
None	34 (6.3)	44 (28)	<.0001

Data are presented as n (%)

*CPAP* continuous positive airway pressure, *HF* heart failure, *NIV* non-invasive ventilation

^a^Data are presented as n (%) or median (IQR) and are among patients who underwent the relevant test and had non-missing data

Echocardiography was performed in 104 patients (15 %) of which 19 have received pulmonary echography. Cardiologists conducted 60 % of echographies while emergency physicians performed 40 % of them (Table 4).

Echographies performed by cardiologists were more likely to be self-rated as satisfactory than those by emergency physicians. Left ventricular ejection fraction (LVEF) did not differ significantly between those with and without HF.

Patients with HF were more likely to receive furosemide, oxygen, and nitrates, but other emergency measures occurred similarly in both groups (Table 4).

Among the 158 patients in whom the diagnosis of HF was not retained, emergency physician reported pulmonary disease in 51.3 % of them (mainly pulmonary infection, pulmonary embolism, and chronic obstructive pulmonary disease (COPD) exacerbation), cardiac disease in 29.1 % (mainly acute coronary syndrome), unspecific diagnosis (discomfort, stress, panting or palpitation) in 10.7 %, renal failure in 5.1 % and gastric disease in 3.8 %.

### Outcomes

Most patients with suspected heart failure dyspnea had acute HF (77 %). ED diagnosis (i.e. HF or NHF) was made at the end of hospitalization by consulting the medical record. Precipitating factors were not determined in 43 % of cases (Table 5). The most common determined precipitating factors were infection (25 %) and arrhythmia (15 %).

**Table 5:** Outcomes^a^

	HF (*n* = 537)	Not HF (*n* = 158)	*P*
Precipitating factors			
Unknown	227 (43)	NA	NA
Infection	135 (25)	NA	NA
Rhythm disorder	81 (15)	NA	NA
Hypertension	57 (11)	NA	NA
Non-adherence to treatment	31 (5.8)	NA	NA
Acute coronary syndrome	23 (4.3)	NA	NA
Eating disorder	20 (3.7)	NA	NA
Diabetes decompensation	10 (1.9)	NA	NA
Discharge destination			<.0001
Cardiology	150 (28)	16 (10)	
Geriatric medicine	62 (12)	5 (3.2)	
Other medical unit	105 (20)	45 (29)	
Cardiology intensive care unit	67 (13)	15 (9.5)	
Resuscitation unit	17 (3.2)	8 (5.1)	
ED hospitalization unit	77 (14)	28 (18)	
Back home	28 (5.2)	29 (19)	
Other	28 (5.2)	11 (6.9)	
Destination considered appropriate	405 (76)	123 (78)	.59
Outcome			
In-hospital mortality	30 (5.6)	21 (14)	.0030
Still hospitalized at 30 days	34 (6.8)	4 (3.4)	.13
Length of stay, days	7 (3–12)	5 (1–9)	.00063

*ED* emergency department, *HF* heart failure, *IQR* interquartile range, *NA* not applicable

^a^Data are presented as n (%) or median (IQR). Percentages for whole table are out of patients with non-missing data

After the ED admission, HF patients were most likely to be hospitalized in a cardiology ward, and only 13 % required treatment in an intensive care unit (Table 5). The destination unit was judged by the emergency physician as appropriate in 76 % of cases.

Only 1 % of patients died in the ED. In-hospital mortality was lower among those with versus without HF (5.6 % vs 14 %; *P* = .003), but length of stay was longer (Table 5).

## Discussion

This large French observational survey of acute HF coordinated by emergency medicine investigators has successfully collected epidemiologic, diagnostic, and therapeutic data, from emergency admission to hospital discharge. As such, DeFSSICA provides information on HF presentation and the French pathway of care.

Not surprisingly, patients in DeFSSICA were elderly, with a median age of 83 years. However, DeFSSICA patients were generally older than those in other HF registries from France (Observatoire Français de l’Insuffisance Cardiaque Aiguë [OFICA]) [4], Switzerland (Acute Heart Failure Global Survey of Standard Treatment [ALARM-HF]) [5], Spain (Epidemiology of Acute Heart Failure in Emergency Departments [EAHFE]) [6], and the US (Acute Decompensated Heart Failure National Registry Emergency Module [ADHERE-EM] [7], Organized Program to Initiate Lifesaving Treatment in Hospitalized Patients with Heart Failure [OPTIMIZE-HF]) [8]. Mean ages in these studies ranged from 73 to 79 years (details of these registries can be found in the Additional file 1). Compared with these registries [4–8], patients in DeFSSICA were less likely to have chronic respiratory failure or chronic HF, but more likely to have chronic renal failure (Web Appendix). Compared with the French OFICA study [4], patients in DeFSSICA were more likely to have hypertension (71 % vs 62 %) and atrial fibrillation (45 % vs 38 %). As atrial fibrillation and a rapid heart rate have been closely linked to mortality [9], detection of atrial fibrillation should be considered systematically.

Furosemide, oxygen, and nitrates were the most commonly used treatments in DeFSSICA patients with HF (75, 68, and 19 %, respectively). The limited use of nitrates may be related to the median (IQR) SBP of 140 (121–160) mmHg. However, our use of nitrates was similar to those in the EAHFE [6] (20.7 %) and OPTIMIZE-HF [8] (14.3 %) registries. Only 10 % of our HF patients received non-invasive ventilation or Boussignac continuous positive airway pressure (CPAP), similar to in the OFICA study (12.4 %) [4]. This ventilation assistance improves outcome in acute HF patients, especially those with pulmonary edema [10, 11]. However, it requires patient compliance and cooperation, which is not necessarily obvious in elderly [12, 13].

Current recommendations for diagnostic investigations in patients with suspected HF include first the assessment of HF probability related to the clinical history (history of coronary artery diseases, or hypertension, or use of diuretics…), the physical examination (bilateral ankle oedema, jugular venous dilatation…) and the ECG (Class I, Level C) [14].

If one of these symptoms or abnormal ECG signs are present, the measurement of BNP or NT-proBNP is the second step in the algorithm of the diagnosis (Class IIa, Level C) with also blood chemistry, and complete blood count (Class I, Level C). Finally, comprehensive assessment of patients with HF comprises, chest X-Ray, transthoracic echocardiography (Class I, Level C), and lung ultrasound (Classe IIb, Level C) [14]. However, in DeFSSICA, only 15 % of all patients underwent echography, due to a lack of equipment and training of emergency physicians. When echography was performed by a cardiologist (60 % of cases), most considered the results to be satisfactory (66 %), but when it was performed by emergency physicians (40 % of cases), only 21 % considered the results to be satisfactory. Therefore, we have identified a need for additional effort for training emergency physicians and providing equipment, as prompt use of echography in the ED has been shown to improve the diagnosis of HF [15].

In line with guidelines, the proportions of patients who underwent ECG, biological analysis, or chest X-ray were all >90 % in DeFSSICA. Similarly, BNP or pro-BNP was measured in 93 % of patients, compared with 82 % of patients in the OFICA study [4]. Not surprisingly, BNP and pro-BNP values were significantly higher among those with versus without HF in DeFSSICA. Several studies have shown the diagnostic and prognostic values of BNP (and troponin) in HF [16–18]; however, they have limited specificity and may be elevated secondary to a variety of causes [9]. Two recent papers have also suggested that neprilysin [19] and soluble CD146 [20] may have a role in the diagnosis of acute HF. Although BNP may be helpful when the diagnosis of HF is in doubt [21], ultrasound remains the gold standard. [22] The use of ultrasound in the ED has been reported [23] to accelerate the diagnosis of HF and the initiation of treatment, and shorten the length of stay. For example, in this study [24] the use of ultrasound could help early diagnosis and therapy for the patient who may need to transfer to the intensive care unit and reduce the length of stay for in-hospital patients. Several others studies have shown the feasibility of ultrasound in the ED and prehospital emergency management of HF patients [24–27]. As the diagnosis of acute HF remains difficult, ultrasound enables emergency physicians to improve their diagnostic performance, optimize their therapeutic strategy, and improve prognosis [28].. In the DeFSSICA survey, only 19 patients received lung ultrasound (LUS) evaluation in the ED. This result clearly shows that, for now, lung ultrasound is scarcely used by French emergency physicians. This fact is really different from the American or Italian practices where LUS is being used frequently. Lung ultrasound is an excellent differential diagnostic tool for distinguishing cardiac causes from pulmonary causes of dyspnea [29].

Pivetta et al. [30] definitively confirmed the excellent diagnostic performance of lung ultrasound to detect acute HF in an ED setting. In their multicenter study that included 1005 patients admitted with acute dyspnea, LUS sensitivity and specificity were respectively 97 % [95 % CI, 95–98.3 %] and 97.4 % [95 % CI, 95.7–98.6 %], and were superior to the one observed with clinical evaluation, chest X-ray and natriuretic peptides. Importantly, the net reclassification index of the LUS-implemented approach compared with standard workup was 19.1 % [30], which suggest the high diagnostic value of LUS in this setting. This performance of LUS has been confirmed in a prehospital setting by Prosen et al. (100 % sensitivity, 95 % specificity) [31]. So it’s probably a key to extend the training facilities for LUS in Europe, especially in France.

To date, the HF pathway of care has been poorly examined in the literature, in particular, patient destinations after ED discharge. It has been suggested that up to half of patients with HF may not require hospitalization [32]. However, in DeFSSICA, 95 % of HF patients were hospitalized, most commonly (28 %) in cardiology units. Approximately 14 % of HF patients were moved to ED hospitalization units, which provide close observation in the absence of a bed in the appropriate unit, or for patients due to be discharged the following day. Over one third of HF patients were hospitalized in non-cardiology units, particularly geriatric units (12 %). If the demonstration was made that the management of HF patients is more optimal in cardiology units [3], the choice of another destination may have been governed by a lack of beds in cardiology and/or the advanced age of some patients requiring a geriatric unit. Considering that majority of patients with AHF should be hospitalized in cardiology or ICU, we asked emergency physicians to assess whether the final destination was appropriate. In more than 75 % of cases they considered it adapted based on the diagnosis and the patient’s clinical status in ED. In 24 % of cases, the ED physician considered that the destination unit was not appropriate, mainly due to lack of beds availability in the unit perceived as appropriate.

In DeFSSICA, 18 % of HF patients had creatinine clearance <30 mL/min. Close cooperation with nephrologists is necessary in these patients, to determine whether dialysis is indicated [28, 33].

Although the incidence of HF decreased between 2000 and 2010 in relation to improvements in the management of myocardial infarction. Regarding to mortality, we have different data between Europe and the United States. While the mortality rate appears stable in the United States between the years 2000-2010 [34], there is a decrease between the years 1987–2008 in Europe [35]. In-hospital mortality of HF patients in DeFSSICA was 6.4 %, slightly lower than in the OFICA study [4] (8.2 %). At 30 days, 6.8 % of DeFSSICA HF patients were still hospitalized, while the median (IQR) length of stay was 7 (3–12) days. In the same idea, the length of stay in DeFSSICA is 7 days in median, like in EAHFE registry [3] and ALARM-HF [5] study which include the patients from the Emergency Department. However in OFICA [4] registry the length of stay is twice. This is probably explained by the inclusion of the patients admitted in intensive cardiac unit who are probably more serious.

Improved interdisciplinary cooperation has been highlighted as a key factor for the improvement of HF patient care [36]. An acute HF syndromes clinical trials network has been set up to improve clinical trials in this patient population [37]. Telemedicine [38] and wireless monitoring could also offer opportunities for improved HF management.

### Limitations

First, the DeFSSICA study was focused only on patients in the ED. Additional prehospital data would be very valuable.

Our study has the strength and the limitation regarding internal and external validity of its multicenter national design. We believe that data collection at different settings, including academic, community, and regional hospitals, makes the result of this study largely applicable to the French healthcare system.

We acknowledge that BNP was not available in every patient despite the IIa European recommendation [14], which is suboptimal. However, it is likely to reflect the “real-life” use of BNP.

One of the limitations of our survey is that we only included dyspneic patients with suspected HF origin, instead of considering all dyspneic patients admitted to the ED. Thus, we found that 23 % of patients don’t have HF.

Finally, a limitation of this survey is that the discharge summary was not subject to expertise for the final diagnosis.

## Conclusion

In conclusion, DeFSSICA has provided insights into the characteristics, diagnosis, treatment pathway, and outcomes of patients with acute HF in French emergency departments. As the diagnosis of AHF remains difficult, Ultrasound enables emergency physicians to improve their diagnostic performance, optimize their therapeutic strategy and improve prognosis. Our study highlights the need for the development of networks, such as those that exist for acute coronary syndromes, for improving the implementation of guidelines.

## Additional file

Additional file 1: Comparison of DeFSSICA survey and data of the main registers of heart failure. (DOCX 17.5 kb)

## Abbreviations

ACEIs: Angiotensin-converting enzyme inhibitors
ADHERE-EM: Acute Decompensated Heart Failure National Registry Emergency Module
ALARM-HF: Acute Heart Failure Global Survey of Standard Treatment
ARBs: Angiotensin II receptor blockers
BNP: B-type natriuretic peptide
CCTIRS: Comité Consultatif sur le Traitement de l’Information en matière de Recherche dans le domaine de la Santé
CNIL: Commission Nationale de l’Informatique et des Libertés (National Commission for Liberties and Data Protection)
COPD: Chronic obstructive pulmonary disease
CPAP: Continuous positive airway pressure
CRF: Case report form
DeFSSICA: Description de la Filière de Soins dans les Syndromes d’Insuffisance Cardiaque Aigue
EAHFE: Epidemiology of Acute Heart Failure in Emergency Departments
ED: Emergency Department
HF: Heart failure
IQRs: Interquartile ranges
LBBB: Left bundle branch block
LVEF: Left ventricular ejection fraction
OFICA: Observatoire Français de l’Insuffisance Cardiaque Aiguë
OPTIMIZE-HF: Organized Program to Initiate Lifesaving Treatment in Hospitalized Patients with Heart Failure
RESCUe: Réseau Cardiologie Urgences (emergency cardiovascular network)
SAMU: Medical dispatch center (Service d’Aide Médicale Urgente)
SFC: Société Française de Cardiologie (French Society of Cardiology)
SFMU: Société Française de Médecine d’Urgence (French Society of Emergency Medicine)
