# Supplementary material for: Management of suspected acute heart failure dyspnea in the emergency department: results from the French prospective multicenter DeFSSICA survey
Source: Scand J Trauma Resusc Emerg Med. 2016 Sep 17;24:112. doi: 10.1186/s13049-016-0300-x (PMC5026775; doi:10.1186/s13049-016-0300-x)
Supplement: Additional file 1: — Comparison of DeFSSICA survey and data of the main registers of heart failure. (DOCX 17.5 kb) [file 13049_2016_300_MOESM1_ESM.docx]

Comparison of DeFSSICA survey and data of the main registers of heart failure

|  | **ADHERE-EM** | **OPTIMIZED-HF** | **ALARM-HF** | **OFICA** | **Registre EAHFE** | **DeFSSICA (HF)** |
| --- | --- | --- | --- | --- | --- | --- |
| Number of center | 83 | 259 | 668 | 170 | 29 | 26 |
| Years of study | 2004-5 (21 month) | 2003-4(22 month) | 2006-2007 (6month) | 2009 (one day) | 2007-11 (4 month) | 2014 (3 weeks) |
| Number of patients | 10984 | 48612 | 4953 | 1648 | 5845 | 537 |
| Age | 73 | 73 | 66-70 | 76 | 79 | 83 |
| Femal (%) | 52.2 | 52 | 62.4 | 46 | 56.5 | 56 |
| ***Comorbidities (%)*** |  |  |  |  |  |  |
| Coronary Heart Disease | NU | 46 | 30.7 | 44 | 30.5 | 30 |
| Hypertension | 79 | 71 | 70.2 | 62 | 82.5 | 71 |
| Diabete mellitus | 45 | 41 | 45.3 | 31 | 42.2 | 28 |
| Atrial Fibrillation | 32.5 | 31 | 24.4 | 38 | 47.6 | 45 |
| Chronic renal failure | 8 | 19.6 | 21.4 | 15 | 22.3 | 28 |
| Chronic respiratory failure | 33.5 | 28 | 24.8 | 20.9 | 24.5 | 17 |
| Chronic heart failure | 76 | 88 | 63.8 | 72 | 63.2 | 54 |
| ***Medications before (%)*** |  |  |  |  |  |  |
| Diuretics | NU | 65.7 | NU | NU | 67.2 | 59 |
| ACE-I / ARB | NU | 51.3 | NU | NU | 55.9 | 45 |
| β-Blocker | NU | 53.1 | NU | NU | 32.7 | 43 |
| Digoxin | NU | 23.4 | NU | NU | 19.7 | 8 |
| ***Clinical abd biological Status(%)*** |  |  |  |  |  |  |
| Cardiogenic Shock | NU | Nu | 11.7 | 6 | 0.7 | 3 |
| Acute Pulmonary edema | NU | Nu | 36.7 | 38 | 11 | 18 |
| SBP* / DBP** | NU | 143/NU | 130/NU | 130/NU | 142/NU | 140/75 |
| Heart Rate*** | NU | 89 | 107 | 89 | 90 | 85 |
| Hyponatremia (%) < 130 mmEq/l | NU | NU | NU | NU | 5.9 | 7% |
| BNP Pro-BNP measured(%) | NU | 8 | Nu | 82 | 34 | 91 |
| ***Emergency therapy (%)*** |  |  |  |  |  |  |
| NIV / CPAP | NU | Nu | 9.6 | 12.4 | 6.4 | 10 |
| Diuretics | NU | Nu | 89.7 | Nu | 96.8 | 75 |
| Vasodilatators | NU | 14.3 | 41.1 | Nu | 20.7 | 19 |
| Inotropics agents | NU | 10.9 | 39 | 13.8 | 2.1 | 1 |
| ***Outcomes - Pathway*** |  |  |  |  |  |  |
| % hospitalized | NU | NU | NU | NU | NU | 90 |
| Length of stay, median | NU | 4-5.7 | 6 | 13 | 7-9.4 | 7(3-12) |
| ICU (%) | NU | Nu | 17 | 43 | 1.9 | 13% |
| In-hospital mortality | 5.8 | 3.8 | 17.8 | 8.2 | 7.6 | 6% |
| 30-days still hospitalized | NU | NU | NU | NU | NU | 7% |
